# Diabetes Mellitus and Mental Handicap—A Preliminary Report

**Published:** 1986-08

**Authors:** J Jancar, R W Lansdall Welfare

**Affiliations:** Consultant Psychiatrist and Clinical Lecturer Stoke Park Hospital, Bristol, and University of Bristol; Senior Registrar and Lecturer Stoke Park Hospital, Bristol, and University of Bristol


### Diabetes Mellitus and Mental Handicap—A Preliminary Report

**J Jancar, MB, BCh, BAO, FRCPsych, DPM**
Consultant Psychiatrist and Clinical Lecturer
**R W Lansdall Welfare, MB, ChB, MRCPsych**
Senior Registrar and Lecturer
Stoke Park Hospital, Bristol, and University of Bristol

When studying the increased longevity of the mentally handicapped (1) we noted a number of cases of diabetes mellitus. We therefore decided to survey the total population of the Stoke Park Group of Hospitals in Bristol resident on 1 August 1982. There were 1,116 mentally handicapped patients of whom 23 were diabetic. (Thirteen females and ten males). At the time of the diagnosis the age range was from 24 years to 75 years with a mean of 56.7 years. The IQ scores ranged from 20–55 with a mean of 34.6.

In reviewing the notes fourteen cases of diabetes mellitus had been diagnosed subsequent to an annual routine urinalysis. Recurrent infections led to diagnosis in five cases and weight loss in the remaining four. Complications of diabetes were identified in 34% of patients. These included repeated infections, hypertension, blepharitis, cataracts, retinopathy and neuropathy. In five there was a family history of psychiatric disorders and in two of diabetes mellitus.

There are a number of known syndromes associated with diabetes mellitus eg Down's, Prader-Willi, Klinefelter's, Laurence-Moon-Biedl syndromes and congenital rubella. In our survey diabetes was associated with the following conditions; Down's syndrome, achondroplasia, Potter's syndrome, tetraploidy, familial microcephaly, atrio-septal defect and Dupuytren's disease (2).

Our survey of a selected mentally handicapped hospitalised population indicates that the prevalence of diabetes mellitus appears to be higher in the mentally handicapped than the general population (3–4).

Diabetes mellitus is believed to represent an heterogeneous group of conditions characterized by a chronically elevated blood glucose. Clinically, there are two forms, insulin dependent, (IDDM) which is usually of juvenile onset and non-insulin dependant (NIDDM) which tends to occur in later life. It is thought that these two forms represent different genetic, hormonal and immunological entities (5). Our survey shows three IDDM and 20 NIDDM patients. To date there has been little research into diabetes amongst the mentally handicapped. With their increasing longevity it is important that it is detected early and proper treatment instituted.

Results of further detailed study of diabetes mellitus, currently being undertaken in our hospitals, will be reported on at a later date.

### REFERENCES

1. CARTER G, Jancar J. Mortality in the mentally handicapped: a 50 year survey at the Stoke Park Group of Hospitals (1930–1980.) J Ment Defic Res 1983 **27**, 143–156.
2. JANCAR J, Griffiths H E D, Sawdon Barbara. Dupuytren's Disease and Mental Handicap. Brit J Psychiat 1985, **147**, 211–212.
3. MALINS F. Glucose tolerance and the diabetic population. Postgrad Med J 1974, **50**, 529–537.
4. FALCONER D S, Duncan L P J, Smith C. A statistical and genetic study of diabetes: Prevalence and mortality. Ann Hum Gen 1971 **34** 347–369.
5. IRVINE W J ed. Immunology of Diabetes. Edinburgh Scientific 1980.

